# Tracking Patterns of Enteric Illnesses in Populations and Communities

**DOI:** 10.1289/ehp.9199

**Published:** 2006-09-14

**Authors:** Terry Peace, Asit Mazumder

**Affiliations:** Water and Watershed Research Program, Department of Biology, University of Victoria, Victoria, British Columbia, Canada

**Keywords:** diagnostic code, drinking water, enteric illness, fee item data, Medical Services Plan, methods for tracking enteric disease, waterborne disease

## Abstract

**Background:**

Enteric illness arising from contaminated water and food is a major health concern worldwide, and tracking the incidences and severity of outbreaks is still a challenging task. Most developed and developing countries have administrative databases for medical visits and services maintained by the government and/or health insurance authorities. Although these databases could be extremely valuable resources to track patterns of environmental and other health issues, test hypotheses, and develop epidemiologic models and predictions, very little research has been done to develop methods to ensure the robustness of such databases and to demonstrate their utility as a research tool.

**Objectives:**

We used the Medical Services Plan (MSP) database of British Columbia, Canada, to develop innovative ways to use medical billing and fee-for-services data to track long-term patterns of enteric illness at the level of populations and communities.

**Results:**

To illustrate the power and robustness of the method, we provided several examples covering 8 years of data from each of four communities covering a large range of population size. Not only could this method generalize to other diseases for which specific fee item markers can be found, but also it gives results consistent with a known outbreak and yields data patterns, which could not be revealed by the currently used methods. Because diagnostic code and fee item data for medical services are collected by most medical insurance agencies, our method can have global applications for tracking enteric and other illnesses at the level of populations and communities.

Enteric illness associated with microbial contamination of water and food is a global issue (Payment et al. 1997; World Health Organization 2006). Unsustainable population growth, increased loading of pollutants and pathogens into source water, and associated contamination of water and perishable food are some of the major causes of increasing rates of enteric illnesses worldwide (Davies and Mazumder 2003). The Milwaukee tragedy in Wisconsin (MacKenzie et al. 1994) and the Walkerton tragedy in Ontario (Clark et al. 2003) are some of the examples of epidemic outbreaks of enteric illnesses originating from contaminated drinking water. Although these outbreaks were severe enough to be recognized as epidemic situations, many minor outbreaks of enteric illnesses are not recorded or recognized because robust methods of tracking the long-term patterns of enteric illnesses in a given population or community are yet to be fully developed.

Like most government health plans, the British Columbia Medical Services Plan (MSP) maintains an electronic database, Program Monitoring and Education Master File (PME-MAST) of the British Columbia government, consisting of records for every service billed to it, approximately 50,000,000 services per year provided to a population of about 4,000,000 in the Province of British Columbia. Details on fee items covered by British Columbia MSP are indicated in Ministry of Health Services (1985, 1998). Because MSP is the sole provider of medical insurance in British Columbia, and the fee-for-service is the dominant business arrangement, this database amounts to about 90% of the services provided by the physicians or surgeons in the province. In addition, this database includes services provided through Worker’s Compensation and through the Insurance Corporation of British Columbia, and is reasonably accurate since 1990. Thus the database is an extraordinarily comprehensive record of medical services, and as such it is a significant resource to model and track patterns of illnesses.

PME-MAST has been used to track enteric illnesses in the past. Aramini et al. (2000) conducted a statistical analysis relating turbidity of water in source water reservoirs to enteric illness using data for the city of Vancouver. PME-MAST was searched for records, which had a diagnostic code related to enteric illness. The authors noted that “the MSP database had only one diagnostic field, a three digit ICD-9 diagnostic field.” We note that the MSP field “diagnostic code” is based on the *International Classification of Diseases, 9th Revision* (ICD-9; World Health Organization 1975), but there are important differences; for example, the ICD-9 code includes a decimal point, whereas the MSP field does not. This renders interpretation of diagnostic code difficult. Aramini et al. (2000) also conducted elementary filtering using “claim specialty” and “service code.” Roslin et al. (2000) also indicated the use of ICD-9 codes in British Columbia in drinking water studies, but did not discuss the limitations of the data. Reid et al. (2001) examined Adjusted Clinical Group methods for predicting health care utilization. This study was innovative in its use of MSP data, beginning the analysis by removing neonates and those with health care service interruptions. They also used age, sex, and the diagnostic code as recorded on the claims, and noted that these data did not include about 8% of the physician services because they were not recorded in the fee-for-service system. Diagnoses from laboratory and diagnostic imaging were not used. Diagnostic code data were augmented by hospital separation abstracts for in-patient admissions and day surgery procedures for the Manitoba database, but not for the British Columbia database. Thus, the authors implicitly noted that diagnostic code data on claims should be augmented by another data source, where possible (Reid et al. 2001). These precautions were taken despite the fact that the study involves all available services, not just a small subset, such as enteric illness. The extent of manipulation we suggest in this article would be wholly impractical in such a case.

Of the three pieces of data of primary interest to epidemiologists (date of service, symptoms, and location of service), only the first field (date of service) is reliable as recorded in the database, because the primary purpose of the database is to pay physicians quickly and accurately, and few data fields are required for this. Even a field as straightforward as physician’s address may refer to home, office, or accountant. Thus the major challenge is to transform this administrative database into an epidemiologic database. Our major objectives in this article are to *a*) develop a set of filters to remove uncertainties inherent in diagnostic code data and location data; *b*) demonstrate how fee item markers can be used to track patterns of enteric illness; and *c*) demonstrate that fee item analysis is significantly more robust than diagnostic code for tracking enteric illness. Given that a computerized database is an emerging and available technology, and given the emerging issues of enteric illness associated with contaminated water and food worldwide, our method would contribute significantly to epidemiologic studies of developing as well as developed countries. This method can generalize to most epidemiologic problems with a fee item marker, in any jurisdiction with a similar set of medical service records.

## Methods

### Filtering of diagnostic codes

It is convenient to infer symptoms from the field “diagnostic code,” often referred to as “ICD-9 code.” ICD-9 codes 001–009 specifically refer to species of enteric illness, but other investigators have included other codes, notably ICD-9 codes 558, 787, and 789. We excluded code 558 because it refers to noninfective inflammation, and we were interested in infective agents. Code 787 refers to symptoms, not infections, so it too was excluded. Code 789 refers to general abdominal and pelvic symptoms; but this would not be used for enteric illness when code 787 was adjacent. Hence code 789 was excluded. This strategy had the virtue of rendering the data set less noisy for enteric illness.

Even with these exclusions, there were complications. Important differences exist among coding systems. ICD-9 codes and its variants use one, two, or three numbers to the left of a decimal, and zero, one, or two numbers to the right of the decimal. But when a claim is submitted to MSP, the decimal point, if present, is removed. Thus, even if physicians and their staffs are perfect in assigning the full range of diagnostic codes, the MSP codes are difficult to interpret, and epidemiologic studies based on MSP diagnostic codes are questionable.

A number of strategies have been proposed. Physicians have been entreated to enter leading zeroes, which distinguish 001, 010, 100, and 100.0. The extent to which this happens now (in 2006) is debatable, but the historical record is unambiguously negative. The approach recommended by MSP (Hu 1996a) is to assume that “all diagnostic codes ... are left justified with no blanks in front of the codes.” Hu asserted that this is valid because “paid services and amounts associated with the matched three digit numerical codes, and codes with L, X, V and Z characters amount to > 98% of the total services and amounts associated respectively with all these codes” (Hu 1996a). To illustrate this, simply recode all entries as 001, the code for cholera. Then all entries are valid ICD-9 and MSP codes, but almost none of the entries match the actual illness. Hu (1996a) reports a quick fix, which in some circumstances may render some of the data interpretable, and thus may be used in the general case (e.g., Reid 2001). But in the case of an epidemiologic study, the situation is quite different because usually only one illness, or one location, or one subpopulation is under consideration at a time. Chaudry (2001) showed how only a few miscoding practitioners created an “epidemic” in one of the largest cities of British Columbia. This discovery prompted her to propose an addendum to the official data dictionary created by one of the authors of this article (T.P.), but to our knowledge this proposal has not been implemented. Further, about 60 diagnostic codes are used by MSP that are not in the ICD-9 lexicon, such as xya, where x and y are digits, and “a” is an alphabetical character. Thus a further challenge to developing epidemiologic methods to track illnesses using medical services data is to filter for these nonstandard diagnostic codes. By filtering the data we mean excluding records according to a specified criterion.

A cursory examination of the data shows that most of the five-digit diagnostic codes that appear in MSP records cannot be readily transformed into valid codes, let alone codes that are consistent with the fee items. Therefore, all of these should be excised. The four-digit codes and five-digit codes are relatively rare, and so the records of practitioners who code these excessively should be treated with skepticism. The same is also true of practitioners who tend to overuse a diagnostic code, so these records were likewise excised. All of the above considerations lead inexorably to the conclusion that, for epidemiologic studies, an alternative to diagnostic code must be found. If this alternative to diagnostic code can be shown to have greater (e.g., *a priori*) validity, it must be the preferred method.

### Integration of diagnostic codes and fee item data into a method of tracking enteric illnesses

To pay physicians quickly and accurately—the administrative purpose for this administrative database—requires few fields, but “fee item” is one of them. Unless this field is correct, the physician will not be paid appropriately. Inappropriate billing and payment may lead to loss of income or investigation. Studies in British Columbia show that intentional misuse is rare. We may therefore have far more confidence in fee item than in diagnostic code. Given the reliability and precision of this field, it is surprising that it has been used only once before to examine enteric illness. The previous use of this approach addressed enteric illness in one community (Hu 1996b), but did not attempt to examine the robustness of the approach with multiple communities. In addition, this approach did not compare diagnostic code data with fee item data, and did not apply the suites of filters used here.

The fee item approach depends on the observation that a subset of the cases of enteric illness has an unambiguous fee item marker. There is a laboratory fee item for each laboratory test, which is used, for example, when the physician attempts to categorize the type of enteric illness from stool samples. In these cases, enteric illness is not merely a preliminary diagnosis. Enteric illness has been observed, and it is only the particular species (pathogen) that is in question. This provides an exceptionally robust set of data, which can only be obtained by fee item. Although it is possible that physicians will be more likely to order tests in the midst of an outbreak, this depends first on the physicians’ arriving at such a consensus. Further, tests would not be ordered for anyone not exhibiting enteric symptom. Therefore, a substantial increase in the number of tests ordered almost certainly corresponds to an outbreak. When the data are filtered using all the filters described above, the data obtained from diagnostic code are nearly disjoint from the data obtained by fee item, which means that diagnostic code data, even when extensively filtered, must be treated with extreme caution.

### Correction of data for location of services

Location data are less problematic than diagnostic code data, but needed further attention in our attempt to make the method more robust. MSP changes the subscriber’s address when it is notified of a move. However, the adjustment or correction of MSP data for mobility is the essential difficulty. Subscribers may seek medical attention while they are on holiday, or may travel to see a specialist or a noted practitioner outside their locality. They may seek attention shortly after moving, or may bring preexisting health conditions with them, and may seek treatment on return to home locations. This suggests removing a record if the practitioner’s postal code or ZIP code is not in the same geographic region as the subscriber’s postal code. However, it is essential to be clear about the exact hypotheses under test, because one may be interested in subscribers from one water system, whereas the practitioners may have practices, or residences, or accountants outside that community. Although limited, this filter will work for primary care, but will not function as intended for secondary or tertiary care. Nor does the filter work for laboratory fee items, because fees in these cases may go to a central payee at a central address. In this case, the postal code of the referring practitioner should be used instead of the postal code of the practitioner providing the service.

Further, young people pose their own challenge for analysis, because young people tend to be very mobile. Mobility exposes them to a greater variety of pathogens than would be found in the general population. Indeed, the concept of “an address,” let alone a known correct address, may be problematic. Our solution was to exclude subscribers 20–29 years of age from the analyses, which accounted for about an eighth of all the records. The problem of relocation may be solved by another set of filters, which remove all subscribers who are unlikely to notify MSP of a change of address as well as those who indicate a recent change of address. The filter method to correct for relocation removed about 1.5% of records from our analyses. Although the number of usable records was somewhat reduced, these filtering methods significantly improved the power our method to track enteric illness in selected populations.

### Computer and statistical analyses

Computing was done on a Compaq Alpha-server (Hewlett Packard, Palo Alto, CA, USA) running Tru64 Unix (Hewlett Packard) and SAS version 8.2 (SAS Institute Inc., Cary, NC, USA). Once the data set for a given population was filtered using the criteria described above, we conducted time-series analyses to examine the patterns of enteric illness over an 8-year period. MSP data are cyclic on a weekly basis because of weekends, so moving average windows were multiples of 7 days.

Removal of these data might have introduced bias. Whether or not a bias is introduced depends on the disease process under consideration, hence the decision to use any given filter must be made on a case-by-case basis. For enteric illness, which poses a greater risk to the compromised immune systems of the very old and the very young, no bias appears to be introduced by any of the filters suggested.

**Figure 1:**
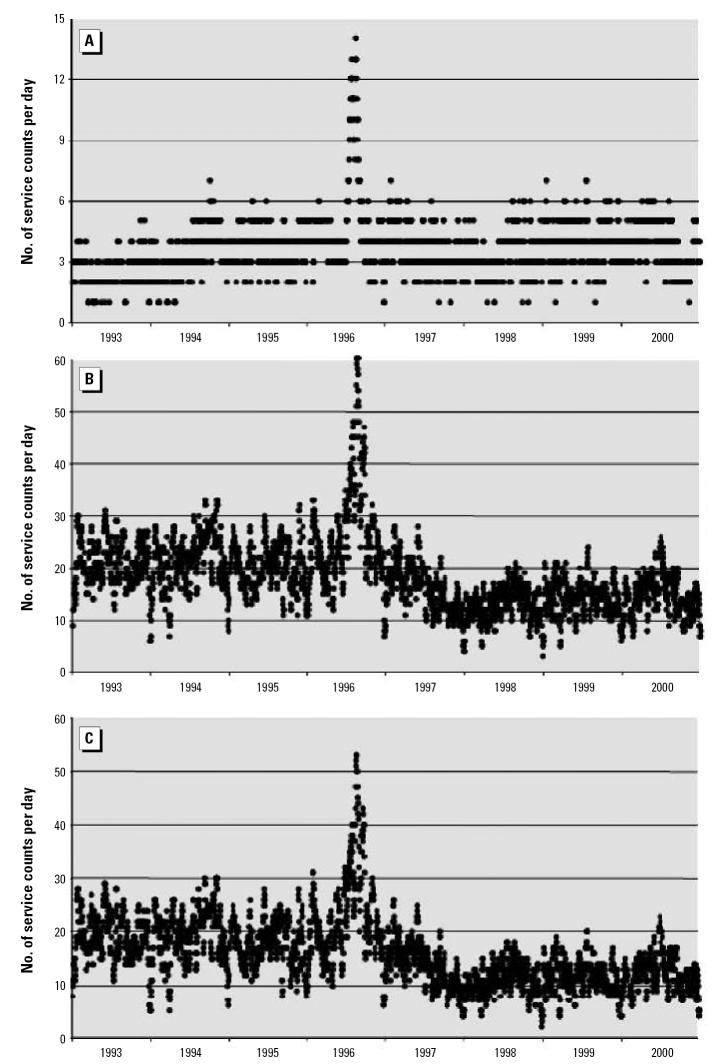
Moving averages (7-day) of services by date for Community 1, with a known epidemic outbreak of enteric illnesses from microbial contamination of drinking water. (*A*) Patterns developed from treatment of data for diagnostic code with basic filtering. (*B*) Patterns developed from treatment of data for diagnostic code and fee item after full filtering. (*C*) Patterns developed from treatment of data for fee item and after filtering.

**Figure 2:**
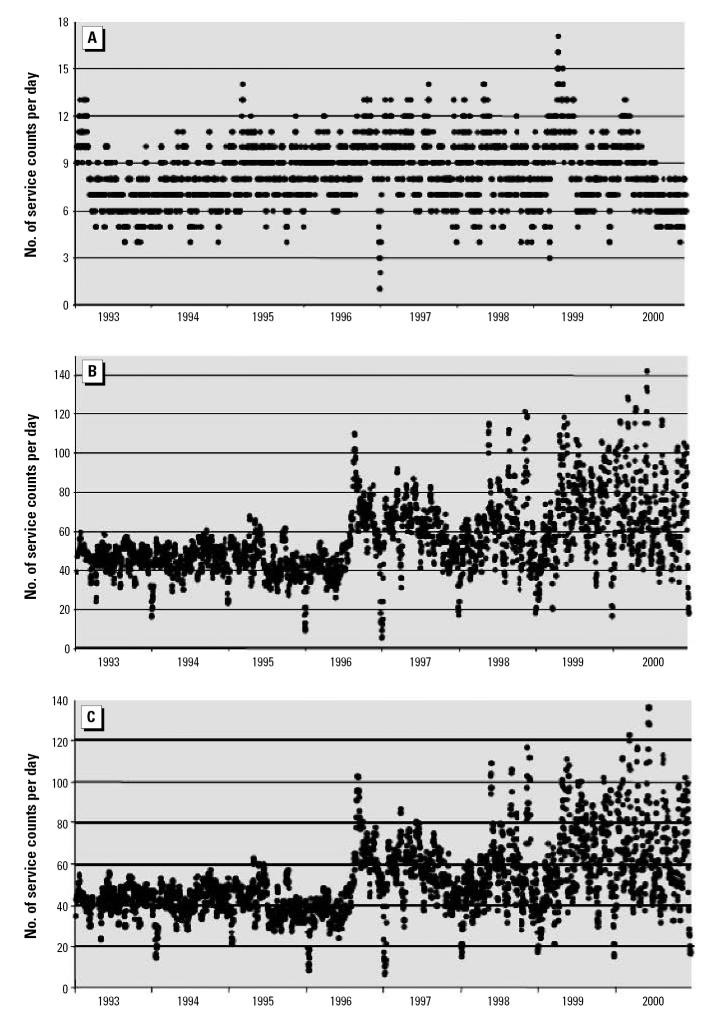
Moving averages (7-day) of services by date for Community 2. (*A*) Patterns developed from treatment of data for diagnostic code with basic filtering. (*B*) Patterns developed from treatment of data for diagnostic code and fee item after full filtering. (*C*) Patterns developed from treatment of data for fee item after full filtering.

**Figure 3:**
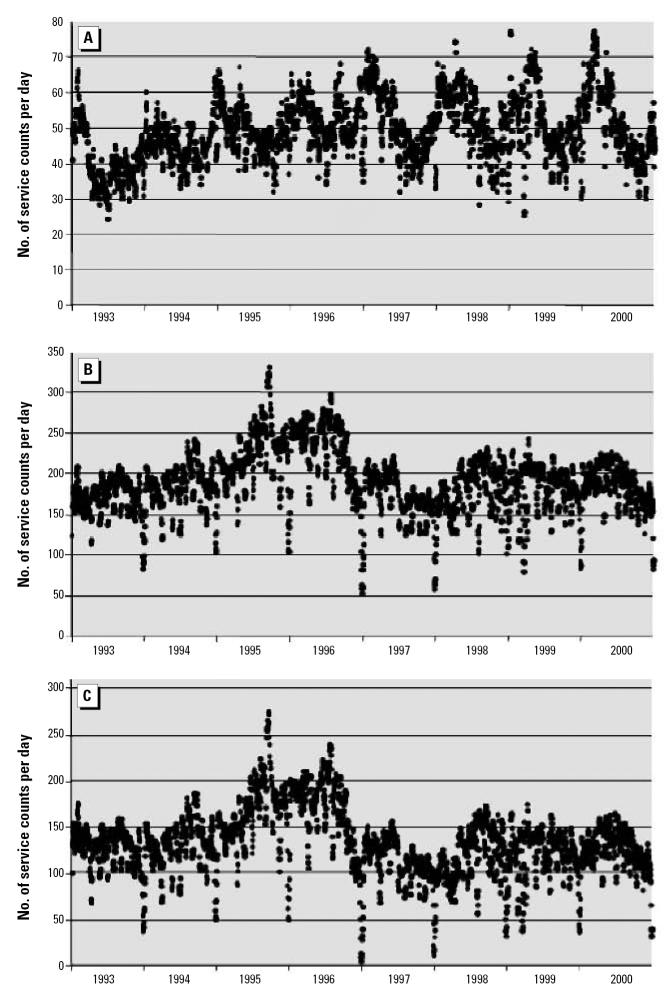
Moving averages (7-day) of services by date for Community 3. (*A*) Patterns developed from treatment of data for diagnostic code with basic filtering. (*B*) Patterns developed from treatment of data for diagnostic code and fee item after full filtering. (*C*) Patterns developed from treatment of data for fee item after full filtering.

**Figure 4:**
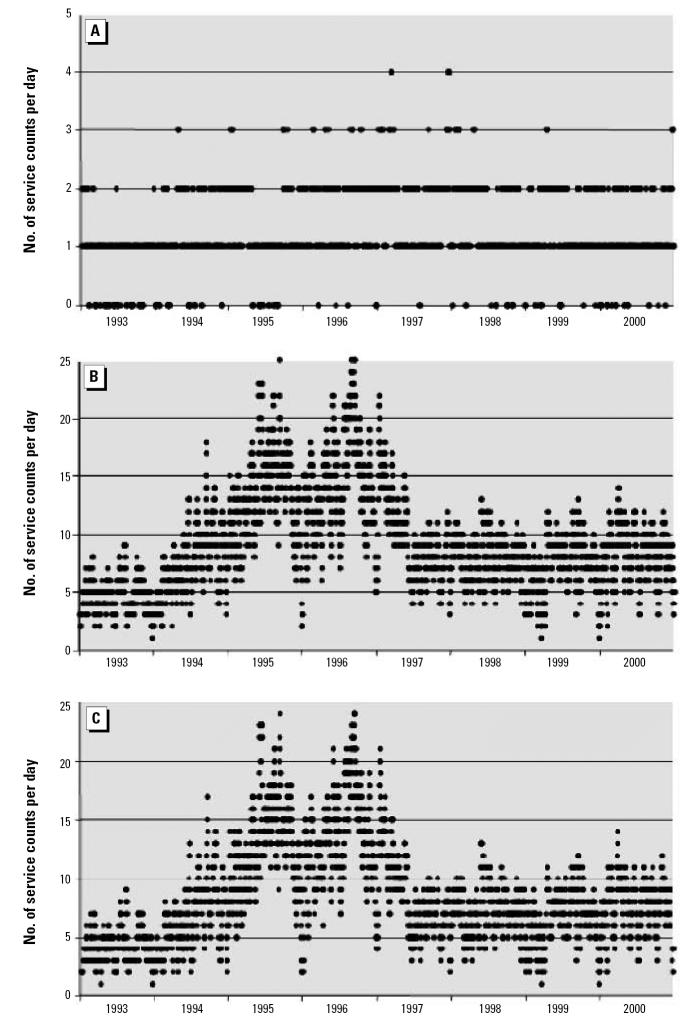
Moving averages (7-day) of services by date for Community 4. (*A*) Patterns developed from treatment of data for diagnostic code with basic filtering. (*B*) Patterns developed from treatment of data for diagnostic code and fee item after full filtering. (*C*) Patterns developed from treatment of data for fee item after full filtering.

As noted above, the analysis based on fee item is inherently more credible, and no specific epidemiologic hypotheses were under consideration. Although we confined ourselves to graphic illustration of the time series of the data and demonstrated remarkable differences in patterns emerging from the application of our methods, we provide below several statistical examinations of the observed differences within each of the four communities. Using the analytic relationship that the standard deviation must be equal to or less than the mean for non-negative data and conservative distributional assumptions, the analysis proceeds with panels A of Figures 1–4 being treated as null hypotheses as for a Monte Carlo. Compare Figures 1A and 1B. Figure 1B reports about four times the number of observations as Figure 1A. For most of Figure 1A, the standard deviation of the observations could not exceed 4 because the mean is at most 4, and the data consist of counts, which are non-negative. For the peak, the standard deviation could not exceed 14 because that is the maximum, which is greater than the standard deviation regardless of the distribution. Nevertheless, take the mean to have a value of 14. Then all the several thousand points on the entire graph of Figure 1B are three standard deviations out, and each observation is significantly different from its counterpart in Figure 1A. Thus, with these highly conservative assumptions, the difference between these two graphics is highly statistically significant. Indeed, assuming normality and no more than 20 independent observations over an 8-year period, statistical significance is such that one would do better trying to guess the exact location of an arbitrary water molecule in the Pacific Ocean. The same argument can be made for Figure 2; further, for Figure 2A, variance appears more or less constant, whereas for Figure 2B, variance rises between 1993 and 2000; *F* = 10 > 1.94 = *F* (df = 250, df = 250; percentile, 0.999). For Figure 3, an argument similar to that for Figure 1 can also be developed. The mean value in Figure 3A is about 40, so the standard deviation cannot exceed 40. In particular, that situation obtains for the 1995 peak of about 340 (Figure 3B), which has a probability no more than one in a trillion for each such value (assuming normality). Observing multiple values not only confirms the validity of the observation, but reduces the *p*-value to the vanishing point, without considering the other few thousand values, which are also larger by at least two standard deviations (each). That the cyclic pattern must also disappear by random processes lowers the *p*-value to the vanishing point and beyond. For Figure 4, the same argument applies, only more forcefully. Four moving averages were considered: 7 days, 14 days, 21 days, and 28 days. All gave similar results, so we chose the 7-day moving average as most revealing. We confined ourselves to an 8-year window for technical reasons.

The four communities under consideration are among the largest in the province. Two have wholly or partially protected watersheds, and now own them entirely. Another community takes its water from deep wells, and is said to have remarkably good water. The final community takes its drinking water from a surface source, and is the site of a laboratory-confirmed outbreak of enteric illness. Two of these four communities are on the Pacific coast and two are hundreds of kilometers inland. Two communities are noted retirement and tourist centers, whereas two are industrial/commercial hubs. These characteristics provide a large variation in conditions, which tend to validate the general application of the methodology. However, in some cases, such as small communities, the method is of limited application, because physicians may be loath to ask their patients to submit stool samples to a remote laboratory.

## Results

To evaluate the method, we considered time-series data from four of the large cities. Because the point of interest here is the efficacy of an approach, only qualitative notions are discussed. We contend that the recommended approach is efficient in preventing both type 1 and type 2 errors, and that this is entirely obvious from the figures. Indeed, when fee item analyses are compared with diagnostic code analyses, opposite trends and conclusions emerge. Hence, mere levels of statistical significance are irrelevant, although, as indicated above, they are very high.

Community 1 drew its drinking water from an unprotected surface source, and experienced a laboratory-confirmed outbreak of enteric illness in June 1996. Figure 1A shows the time series after basic filtering, which consists of all the diagnostic code filters noted and some of the other techniques discussed in “Methods.” The peak of services in the middle of 1996 was obviously related to an outbreak of enteric illness resulting from contaminated source water.

Figure 1A gives the distinct impression that the outbreak is a unique event and that enteric illness appears to be trending upwards. The number of services is not large and, considering the population, it is worrisome. From the graph it is impossible to say whether the public health is particularly good, or whether most physicians are reluctant to code office visits as enteric illness. A higher level of filtering and inclusion of the laboratory fee items show more robust patterns.

In Figure 1B, the number of records has risen by an order of magnitude, giving us much more confidence in the data. It is therefore clear that in this community, physicians appear to be reluctant to code office visits as enteric illness, which must be cautionary for those who rely on diagnostic code for evidence of disease. The outbreak is clearly visible, but it is clear from the high background incidence that the outbreak did not erupt from a vacuum. After the outbreak, the baseline number of services drops dramatically, suggesting preventive measures taken by officials, the public, or both. An examination of laboratory items by themselves reinforces these observations. In Figure 1C, note particularly the downward trend since the outbreak. The background is rather lower than it was before, and has not rebounded. This is a very hopeful sign that something has been accomplished to prevent another outbreak. Such an observation is not possible in current published methods using diagnostic codes alone (Figure 1A); thus a type 2 error has been avoided, with a probability of 1.

Community 2 draws its water from a more or less protected watershed. Most parts of the watershed had been fully protected before 1995. Now the entire watershed is protected with enhanced security preventing any public access. Figure 2A shows the services based on diagnostic code with basic filtering of data, which indicates a minor peak in services during mid 1999. This does not correspond to any known outbreak. Further, there is an increasing trend in the data until 2000, followed by a declining trend. The addition of fee item data does more than just increase the number of services; it significantly changes the time-series patterns of enteric illnesses. The 1999 peak of Figure 2A disappears, minor peaks appear in the middle of the decade, and incidences of enteric illness become highly variable. There is no overall trend in the minima, but the maxima trend upward. In Figure 2C, our use of fee item alone does not change the picture of Figure 2B. Although the features of Figures 2B and 2C may turn out to be an artifact of the data analysis, the filtering has at least ruled out many obvious sources of error. Analysis by fee item, the gold standard, alerts us to the possible presence of three unobserved effects (mid-1990s peak, increasing maxima, and increasing variability), all of which are invisible to traditional analysis of diagnostic code (Figure 2A). Type 2 errors have been avoided, with a probability of 1.

Community 3 draws its drinking water from a surface source, which has been fully protected for the last several decades. Figure 3A is a graph of services identified by diagnostic code, which shows cyclic tendencies and a generally increasing trend. This data pattern has moved other investigators to seek an explanation for the cyclic tendencies and to note the significant increasing trend of enteric illness. These tendencies are both scientifically interesting and, for the water utility, alarming. However, further filtering and the inclusion of fee item data alter the picture (Figure 3B). This figure indicates clear peaks of services in 1995 and 1996, followed by a return to baseline. The cyclic pattern disappears. The return to baseline is a reassurance, not a call to action. The unsuspected peaks in 1995 and 1996 might be interesting from public health perspectives, to seek for potential causes. However, the two time-series patterns are wholly distinct, both from a scientific and a public policy perspective. Figure 3C shows the pattern of enteric illness revealed by the use of fee item data alone, and further emphasizes the return to baseline in late 2000. It is unmistakably clear that inclusion of fee item data fundamentally alters the situation. There is no longer a motive to seek cyclic correlates to enteric illness, and a major type 1 error has been avoided. An opportunity to explain the peaks of 1995 and 1996 also arises, and a type 2 error is likewise avoided.

Community 4 draws its drinking water from deep wells, and is generally considered to have some of the finest drinking water in the province. The traditional method, using diagnostic code, shows no consistent pattern of enteric illness in this community (Figure 4A); indeed, it hardly registers. Inclusion of fee item data changes the pattern dramatically (Figure 4B). Peaks in the summers of 1995 and 1996 rise to nearly 30 services per day, which deserve further investigation of this pattern with other sources of relevant data from the water utility. Given the view that this source water is of very high quality, even the baseline of 12–15 services of Figure 4B is surprising. Consideration of superior data—data that have been filtered using the method presented in this paper—is leading us to quite different conclusions about the incidence of enteric illness, and enticing us to seek explanations. Fee item data are considered on their own in Figure 4C. Again, we see the peaks during the summers of 1995 and 1996. Although the diagnostic code data can be dismissed as random, the peaks shown by fee item data suggest the possibility of real outbreaks of enteric illnesses, which may not have been recognized by public health officers. Again, a major type 2 error has been avoided.

## Discussion

Our results show that an analysis based on fee item is far superior to the analysis of enteric illnesses based on diagnostic code. Therefore, an analysis based on diagnostic code should be viewed with caution until it is either validated by an analysis based on fee item, or an analysis based on fee item is found to be impractical. As noted above, this situation may exist when the community is too small to support a medical laboratory or when the community is isolated by distance or by weather, or some other local conditions. In such a case, it is all the more important to use robust filters appropriate for different conditions related to community size and availability of services.

The proposed method, consisting of both filtration of diagnostic codes and inclusion of fee item data, is inherently more robust than simple reliance on diagnostic code. First, the method is useful in eliminating the most egregious type 1 errors, such as in Community 3, in which an alarming and interesting data pattern, based on diagnostic code only, is shown to misrepresent reality. Not only is the cyclic pattern illusory, but also the increase in services is artifactual. The method is equally useful in avoiding type 2 errors, as in Communities 2 and 4. Community 2 shows a dramatic rise in the variability of enteric services, which points to public health concerns deserving further investigation. The pattern of enteric illness shown in Figure 2C needs further explanation, because this pattern is wholly absent in Figure 2A for the same community. In Community 4, the true levels of enteric illness are wholly unsuspected from consideration of Figure 4A, and a type 2 error is inescapable without reference to fee item data. Finally, the method is useful in improving power by increasing the number of cases, as is demonstrated in Figure 1. Figure 1A indicates a baseline of 4 services, and a peak of 14 services, whereas the addition of fee item data, even after extensive filtration, reveals a baseline of 20 services and a peak of 60 services (7-day moving average). Additionally, Figure 1B avoids the type 1 error of seeing an increasing trend in the services provided, and avoids the type 2 error of not seeing the decreased baseline after the outbreak.

Because these four communities represent some of the largest population centers in the province, some significant bias may be inferred. Not only do these communities have large communities of physicians, but also the communities are well served by laboratory diagnostic services. Both of these features tend to make an analysis based on fee item more reliable than it might be in a small community, where all the physicians interact frequently, and are perhaps less likely to order laboratory diagnostic services, prescribing them only for the most serious complaints. Also, the public in large and small communities may react differently, based partially on the convenience (or inconvenience) of services provided (or not). As with any analysis of administrative data, local conditions should be considered.

Our method and associated results show that the time-series patterns emerging from fee item and filtration can elucidate patterns of enteric illness at the level of communities. This method is a significant improvement over currently published and currently practiced methods of using medical administrative data to track patterns of enteric illnesses. Our method is particularly useful when the diagnostic code field is suspect or is not rigorously checked by the payment agency. Finally, the method developed here can be applied to other health databases where one or more fields in the database are questionable. Because all of the developed and some of the developing nations currently maintain health and medical databases, this method can be used robustly not only to assess patterns of enteric illnesses in a community or a population, but also to help track other environmental or nonenvironmental health risks and scenarios. In this article we focused only on developing robust methods for using medical billing (MSP) and similar databases to track patterns of enteric illness, which can be used effectively to conduct epidemiologic studies at the levels of populations and communities. Future research interest in this area may be directed towards patterns of co-morbidity or linkages among environmental quality and public health and epidemiology.
